# Prevalence of Myofascial Trigger Points in Patients with Mild to Moderate Painful Knee Osteoarthritis: A Secondary Analysis

**DOI:** 10.3390/jcm9082561

**Published:** 2020-08-07

**Authors:** Eleuterio A. Sánchez Romero, Josué Fernández Carnero, Jorge Hugo Villafañe, César Calvo-Lobo, Victoria Ochoa Sáez, Verónica Burgos Caballero, Sofia Laguarta Val, Paolo Pedersini, Daniel Pecos Martín

**Affiliations:** 1 Department of Physiotherapy, Faculty of Biomedical and Health Sciences, Universidad Europea de Madrid, 28670 Madrid, Spain; 2 Musculoskeletal Pain and Motor Control Research Group, Faculty of Health Sciences, Universidad Europea de Madrid, 28670 Madrid, Spain; 3 Department of Physical Therapy, Occupational Therapy, Rehabilitation and Physical Medicine, Rey Juan Carlos University, 28943 Madrid, Spain; sofia.laguarta@urjc.es; 4 La Paz Hospital Institute for Health Research, IdiPAZ, 28922 Madrid, Spain; 5 Grupo Multidisciplinar de Investigación y Tratamiento del Dolor, Grupo de Excelencia Investigadora URJC-Banco de Santander, 28922 Madrid, Spain; 6 IRCCS Fondazione Don Carlo Gnocchi, 20161 Milan, Italy; mail@villafane.it (J.H.V.); pedersini93@gmail.com (P.P.); 7 Faculty of Nursing, Physiotherapy and Podiatry, Complutense University of Madrid, 28040 Madrid, Spain; cescalvo@ucm.es; 8 Older-adult care center "Manuel Herranz", Pozuelo de Alarcón, 28223 Madrid, Spain; vitofisio@hotmail.com (V.O.S.); evdpozuelo.rhb@gmail.com (V.B.C.); 9 Department of Physical Therapy of Alcalá University, Alcalá de Henares, 28805 Madrid, Spain; daniel.pecos@uah.es

**Keywords:** myofascial pain, knee, osteoarthritis

## Abstract

Objective: To determine the prevalence of myofascial trigger points (MTrPs) and the correlation between the number of MTrPs and pain and function in patients presenting knee pain osteoarthritis (OA). Methods: This was a secondary analysis of data from a cross-sectional study. The prevalence of MTrPs located in tensor fasciae latae, hip adductors, hamstrings, quadriceps, gastrocnemius, and popliteus muscles was studied in 114 patients (71 men and 43 women) with knee OA. Pain and functionality were assessed with a numerical pain rating scale (NPRS), the Western Ontario, McMaster Universities Osteoarthritis Index (WOMAC) score, the Barthel Index, and the timed up and go test. Results: The prevalence of latent MTrPs was detected via palpation and was estimated to be 50%, 35%, 25%, 29%, 33%, and 12% for tensor fasciae latae, hip adductors, hamstrings, quadriceps, gastrocnemius, and popliteus muscles, respectively. The prevalence of active MTrPs was estimated to be 11%, 17%, 30%, 18%, 25%, and 17% for tensor fasciae latae, hip adductors, hamstrings, quadriceps, gastrocnemius, and popliteus muscles, respectively. Pain was measured with the NPRS scale and was poorly correlated with the prevalence of latent MTrPs (r = 0.2; *p* = 0.03) and active MTrPs (r = 0.23; *p* = 0.01) in the hamstrings. Disability was moderately correlated with the number of latent MTrPs in the tensor fasciae latae muscle (Barthel, r = 0.26; *p* = 0.01 and WOMAC, r = 0.19; *p* = 0.04). Conclusions: This secondary analysis found that the prevalence of the MTrPs varied from 11% to 50% in different muscles of patients with mild to moderate painful knee osteoarthritis. Pain was correlated poorly with the prevalence of latent and active MTrPs in the hamstring muscles, and disability correlated moderately with the number of latent MTrPs in tensor fasciae latae.

## 1. Introduction

In Europe osteoarthritis (OA) is the most common form of chronic pain (34%) reported, and has a high economic and social burden on society [1]. This burden is growing as the population increases in number and in age. The precise etiology of OA remains thus far unknown, despite identification of various risk factors associated with presence of the disease, including age, sex, obesity, diet, and local joint injury [2]. The population with knee OA progressively experiences an increase in pain and difficulty in performing the tasks of daily life [3]. The overall prevalence was estimated at 3.8%, being higher in women (4.8%) than in men (2.8%) [4]. In Spain it is estimated that 10% of the general population has characteristic symptoms of knee OA [5,6].

Pain-osteoarthritis (pain-OA) has traditionally been viewed as peripherally mediated nociceptive pain [7]. Many people with symptomatic OA report chronic joint pain, especially when the patients are over 50 years of age. The gap between pain-OA and OA is usually explained by the propensity of some OA patients to develop sensitization [8]. It has been suggested that a common musculoskeletal injury can result in a disturbance of the nociceptive system [9], including central sensitization [10], and that conditioned pain modulation is impaired [11,12]. The transition from nociceptive sensitization to more chronic widespread pain can occur due to a repetitive nociceptive event [13,14]. In this scenario, myofascial trigger points (MTrPs) treatment is considered a contributor to improving sensory and motor disturbances in many musculoskeletal disorders. A recent systematic review about the effects of dry needling on the MTrPs in patients with knee pain syndromes revealed that this approach was effective for decreasing pain in patellofemoral pain, but was not with in knee OA or post-surgical knee pain [15].

The presence of MTrPs in muscles near the knee is often ignored during the clinical diagnosis, which may be a primary or secondary cause of another diagnosis [16]. The new MTrPs criteria [17] establish findings during the muscular physical examination, including a taut band, a hypersensitive spot, and referred pain accompanied by different sensory sensations such as tingling, burning, heaviness, and others. Furthermore, a specific location should not be expected for the referred symptoms, differentiating between active and latent MTrPs in the reproduction of any of the symptoms experienced by a patient and the recognition of pain. It is not known whether trigger points can be a cause of pain in knee OA or simply coexist with pain [18]. However, to the best of our knowledge, there is no study in the literature that defines the prevalence of MTrPs in knee OA patients, even if many interventional studies deal with this issue [19,20].

Improvements in world health care have led patients to focus their attention on prevention, seeking care for pain and the correct management to slow down the progression of OA. Identifying the factors that lead to pain is fundamental for improving the management of knee OA symptoms, and preventing the onset of permanent or chronic pain [21]. In this current work, a secondary exploration of our previous cross-sectional study was conducted [22], to determine the prevalence of MTrPs and the correlation between MTrPs and pain and function in patients presenting OA knee pain.

## 2. Methods

### 2.1. Subjects

We performed a secondary analysis of data from a cross-sectional study. The previous study aimed at describing and comparing demographic, clinical, and myofascial pain syndrome characteristics in patients with knee OA by sex and age distribution. The methods and description of the trial have been previously described [22]. The most relevant parts of the design are summarized below.

Subjects with knee pain, unilateral or bilateral dysfunction, and diagnosis of primary knee OA, fulfilling the American College of Rheumatology criteria for clinical and radiographic diagnostics, [23] were eligible for the study (Kellgren and Lawrence scale between 1–3) [24]. The patients were included according to the peak pain intensity over the preceding week, assessed through a numerical pain rating scale (NPRS) (patients with a peak pain of <6 suffering from mild to moderate knee pain) [25]. The chronicity of pain was taken into account, ranging from 8 to 204 weeks from the onset of pain. Patients were excluded from the study if they suffered from any other health condition that could cause myofascial or neuropathic pain in the lower limbs. The inclusion and exclusion criteria were based on previous studies [25,26,27,28,29]. This study was carried out by two physical therapists (EASR and VOS) and one occupational therapist (VBC) with 10 years’ experience in musculoskeletal disorder. One physical therapist (MVOS) carried out the assessments to collect sociodemographic and primary outcome measurements. A second physical therapist (EASR) performed the physical examination to detect active or latent MTrPs in the muscles of the involved lower limb (‘s), using recently updated criteria described by Travell and Simons [17,30].

The current study was approved by the Clinical Research Ethics Committee of the Rey Juan Carlos University, Madrid, Spain (approval number: 13/2015); it was registered on ClinicalTrials.gov (Identifier: NCT02698072) [22]. All patients provided informed consent prior their enrollment [22].

### 2.2. Outcome Measurement

One physiotherapist (MVOS) and the occupational therapist (VBC) carried out pain, function, and test assessments. Results were collected for pain intensity and function using the following tools: NPRS; Western Ontario and McMaster Universities Osteoarthritis Index (WOMAC); the Barthel Index (BI); and the timed up and go test (TUG). Sociodemographic data such as age, sex, and body mass index were collected. Chronicity of knee pain, calculated in months, was also noted. The primary outcome was the palpation intervention, while the secondary ones were pain intensity, function, and test assessments related to the presence of MTrPs in pain-OA patients.

### 2.3. Detection of Active or Latent MTrPs

The tensor fasciae latae, hip adductors, hamstrings, quadriceps, gastrocnemius, and popliteus muscles were examined in each subject following a protocol regarding patient and limb positions, exactly reproduced from the study of Mayoral et al. [31]. These muscles are frequently involved in myofascial knee pain. Presence of active or latent MTrPs was defined on the basis of the palpation criteria reported below:

(1) Is there a taut band (or TB)?
(2) Is there a palpable nodule in the taut band (NE)?
(3) Is there a hypersensitive point (HN)?
(4) Is there referred pain familiar to patient’s pain (RP)?

We had latent MTrPs when there was concurrence of the following 3 criteria: TB, NE, and HN. We had active MTrPs when all the four criteria were present [17,30,32]. Patients were considered according to this syndrome if they had at least one active (pain-generating) MTrP [17,30]. All MTrPs were collected and we were able to determine MTrP prevalence.

### 2.4. Pain Intensity, Function, and Test Assessments

Pain intensity was measured with the 11-point NPRS (intervals from 0–10), where 0 corresponded to no pain and 10 to the worst pain imaginable. The NPRS is a valid and reliable tool, useful in elderly adults [33,34]. The WOMAC score was used to evaluate symptomatology and function, regarding knee OA patients [35,36]. The BI tool was used to quantify everyday life activities [37]. It includes 10 items with a total score ranging from 0–100 points. A higher score corresponds to better capacity for performance of everyday life activities.

In the TUG, patients stood up from a seated position on a standard chair, walked to a line on the floor three meters away, returned to the chair, and sat down again. The score was evaluated by the time taken in seconds to complete the test cut-off point of ≥13.5 [38]. Consecutive time ranges indicated a gradual increase in the risk of falling.

### 2.5. Statistical Analysis

Data were analyzed using SPSS for Windows (V.25, IBM, Armonk, NY, USA). Descriptive statistics (mean and standard deviation) were provided for all the subjects. The relationships between the number of active and latent MTrPs (prevalence) of the six muscles examined (tensor fasciae latae, hip adductors, hamstrings, quadriceps, gastrocnemius, and popliteus muscles) and pain or function were assessed using Pearson’s correlation coefficients. Then, the correlation between the number of trigger points was made, using the requirement of having active and latent trigger points with the clinical variables included in the study. Correlation coefficients with cut-off values of 0.3 and 0.7 were used to distinguish poor/moderate/good correlation.

## 3. Results

### 3.1. Clinical Characteristics of the Participants

One hundred and sixty-five (*n* = 165) consecutive subjects accepted to participate in this study. Seventy-one men and 43 women with knee OA aged from 65 to 86 years (mean, 72.3; SD, 5.3 years) satisfied all eligibility criteria. The reasons for ineligibility were rheumatoid arthritis (*n* = 11) and no confirmation of the diagnosis with radiographs (*n* = 39). Descriptive statistics for demographics, pain, and functional assessments, including mean values of NPRS, WOMAC, BI, and TUG scores, are presented in Table 1.

### 3.2. Prevalence of Latent and Active MTrPs

The prevalence of latent MTrPs was estimated to be 50.0%, 35.1%, 25.4%, 28.9%, 33.3%, and 16.7% for tensor fasciae latae, hip adductors, hamstrings, quadriceps, gastrocnemius, and popliteus muscles, respectively. The prevalence of active MTrPs was estimated to be 10.5%, 16.7%, 29.8%, 18.4%, 25.4%, and 12.3% for tensor fasciae latae, hip adductors, hamstrings, quadriceps, gastrocnemius, and popliteus muscles, respectively. Performance of the test results for the number of latent and active MTrPs are presented in Table 2.

### 3.3. Correlation between MTrPs and Pain and Disability Scores

Pain was measured with the NPRS scale and correlated poorly with the prevalence of latent MTrPs (r = 0.2; *p* = 0.03) and active MTrPs (r = 0.23; *p* = 0.01) in the hamstring muscles. Disability correlated moderately with the number of latent MTrPs in tensor fasciae latae muscle (Barthel, r = 0.26; *p* = 0.01 and WOMAC, r = 0.19; *p* = 0.04). The Pearson’s correlations between pain severity, disability, and the number of MTrPs variables are presented in Table 3.

## 4. Discussion

The current secondary analysis was focused on the prevalence of MTrPs and the correlation between the number of MTrPs and pain and function in patients presenting knee pain OA. The results demonstrated statistically significant correlations, where the hamstrings (30%) had the highest prevalence of active MTrPs. The tensor fasciae latae showed the highest prevalence of latent MTrPs (50%). The analysis of correlation between pain, disability, and the presence of MTrPs was low and moderate for these two muscles, respectively.

There was a significant difference in the total duration in months from the chronicity of pain among participants. However, in the cohort study developed by Neogi et al. [28], analyzing 2126 subjects, researchers investigated the correlation of the mechanical temporal summation and pressure pain thresholds with the duration and severity of the knee OA, using knee X-rays, pain questionnaires, and assessment of factors that can influence pain sensitivity. The authors found no relationship between the onset of pain and the measured variables.

Our results contrast with those of Alburquerque-García et al. [39], where the authors observed an association between the presence of active MTrPs and higher intensity of ongoing knee OA pain. However, since the results of the Alburquerque-García study were based on a limited number of women (*n* = 36), no useful comparison can be made with the results of the present study. Differences in results between our study and that of Alburquerque-Garcia et al. could perhaps be explained by the difference in sample size and/or gender, or that they compared 18 subjects affected by knee OA, diagnosed according to the American College of Rheumatology classification, with 18 asymptomatic subjects. In another observational study [40], also presenting a small sample size (*n* = 28: 14 patients with hip OA, Knee OA, or both, and 14 healthy subjects), a significant positive correlation between the total number of trigger points and their radiological scores was found (Spearman r = 0.57, *p* = 0.04); unilateral knee joint OA patients had a greater number of MTrPs in muscles surrounding the knee joint, compared to the unilateral hip joint OA patients. These results contrast with the ones of the present study. However, due to the limited sample size in the previous study, a risk of bias exists. The authors of the previous study also found that the highest prevalence of MTrPs was observed in the gastrocnemius at 57.1%, whereas in our study, it was less, at 25%. Itoh et al. [41] observed the highest MTrP prevalence in the quadriceps, which contrasted with our observation of high prevalence in the hamstrings.

A previous study evaluating patients who were awaiting total knee arthroplasty surgery [42], found MTrPs in the measurable muscles of 62.5% of patients, with more than a third (37%) presenting pain in the medial and lateral compartment. However, the authors did not explore the relationship between the presence of pain and MTrPs.

Disability correlated moderately with the number of latent MTrPs in the tensor fasciae latae in our study; it should be noted that therapeutic improvements in pain are not always accompanied by improvements in functional parameters, as pointed out by White et al. [43]. In their study, authors observed that in a cohort of 3026 subjects with knee OA, 20% of the sample presented an altered function measured by walking speed, despite having reduced their intensity of knee pain by 41%.

In a previous study, Neogi et al. [44] affirmed that there is a relationship between the synovial membrane inflammation, assessed by magnetic resonance, and the painful sensitization measured by quantitative sensory tests, after examining 1111 subjects affected by knee OA. These findings seem to be in line with those obtained in our study, even if we could not establish a consistent relationship between the peripheral muscular source of pain and pain presented by patients with knee OA.

It has been suggested that the myofascial pain syndrome, as well as central sensitization is at the origin of a persistent nociceptive input [45,46], but the results of our study do not support the theory that myofascial pain is contributory to the alterations of the nociceptive processing suffered by patients with knee OA. Our results, which correlate MTrPs in patients with mild to moderate painful knee osteoarthritis, are contradictory to those found by Niddam et al. [47], since they relate directly the intramuscular electrostimulation on an upper trapezius MTrP to the effect of conditioned pain modulation.

The factors that mostly contribute to the development and progression of knee OA, are diet [48], socioeconomic status [49], and genetics [50]. We consider our epidemiological study as an important contribution to the knee OA prevalence field related to Myofascial Pain Syndrome, despite the limitations of this type of investigation. Successful management of the prevention of OA, as well as pain related to OA, requires epidemiological and interventional studies to support and improve function and reduce pain [51]. 

Different limitations were found in the study. The first and main limitation was the small sample size and the lack of a control group of participants. Another limitation was due to the symptoms of some patients that can worsen, as this behavior is identified as threatening or harmful. However, the present study did not evaluate depression or anxiety variables, sleep efficiency, or catastrophizing.

The current study defined data about the prevalence of latent and active MTrPs in patients with knee OA, and found a poor correlation between pain and the presence of the MTrPs. The MTrPs prevalence points estimated in this study should be viewed with caution and future studies with large samples are needed. As such, we think that our results may open up a new way to investigate temporal pain, or conditioned pain modulation by active or latent MTrPs, to establish the contribution of active or latent trigger points to pain in knee OA. Therefore, there is a clear need for trials examining the potential role of treating MTrPs in patients with knee OA in a multimodal rehabilitative approach.

## 5. Conclusions

This secondary analysis found that the prevalence of MTrPs varied from 11% to 50% in various muscles of patients with mild to moderate painful knee OA. Pain was correlated poorly with the prevalence of latent and active MTrPs in the hamstring muscles, and disability correlated moderately with the number of latent MTrPs in tensor fasciae latae.

## Figures and Tables

**Table 1:** Descriptive statistics and functional scores of the cohort.

Parameter, (*n =* 114)	Continuous and Categorical Variables
Age, (mean, SD)	72.3, ±5.3
Gender, men (*n*, %)	71, 62.3%
Weight, (mean, SD)	75.8, ±11.5
Height, (mean, SD)	158.7, ±0.9
BMI, (mean, SD)	30.1, ±4.6
NPRS, (mean, SD)	6.1, ±1.0
WOMAC, (mean, SD)	33.6, ±11.4
Barthel Index, (mean, SD)	96.1, ±5.1
Timed Up and Go, (mean, SD)	10.3, ±3.3

Abbreviations: SD, standard deviation; %, percentage; NPRS, visual analog scale; BMI, body mass index; NPRS, numerical pain rating scale; WOMAC, Western Ontario and McMaster Universities Osteoarthritis Index.

**Table 2:** Analyses of prevalence of active and latent MTrPs in patients with knee osteoarthritis (OA).

	Number	Percentage (%)
**Latent MTrPs, (TB + NE + HN)**		
Tensor fasciae latae	57	50.0
Hip adductors	40	35.1
Hamstrings	29	25.4
Quadriceps	33	28.9
Gastrocnemius	38	33.3
Popliteus	19	16.7
**Active MTrPs, (TB + NE + HN + RP)**		
Tensor fasciae latae	12	10.5
Hip adductors	19	16.7
Hamstrings	34	29.8
Quadriceps	21	18.4
Gastrocnemius	29	25.4
Popliteus	14	12.3

Abbreviations: MTrPs, myofascial trigger points; TB, taut band; NE, nodule; HN, hypersensitive nodule; and RP, referred pain.

**Table 3:** The Pearson’s correlations between pain severity, disability and the number of Myofascial Trigger Points.

		Active					
		Tensor Fasciae Latae	Hip Adductors	Hamstrings	Quadriceps	Gastrocnemius	Popliteus
**NPRS**	Pearson Correlation	−0.11	−0.01	−0.20 *	−0.03	−0.02	−0.04
	*p*-value	0.26	0.93	0.03	0.75	0.83	0.66
**WOMAC**	Pearson Correlation	0.03	0.07	0.07	0.01	0.17	0.13
	*p*-value	0.76	0.45	0.64	0.90	0.06	0.18
**Barthel Index**	Pearson Correlation	−0.07	0.05	0.01	0.05	0.09	−0.07
	*p*-value	0.46	0.60	0.91	0.57	0.33	0.43
**TUG**	Pearson Correlation	−0.07	−0.06	0.03	−0.06	−0.01	−0.01
	*p*-value	0.49	0.52	0.75	0.52	0.91	0.27
		**Latent**					
**NPRS**	Pearson Correlation	−0.02	−0.09	−0.23 *	−0.02	−0.03	−0.08
	*p*-value	0.84	0.35	0.01	0.81	0.73	0.40
**WOMAC**	Pearson Correlation	0.19 *	0.03	0.03	0.06	0.13	0.11
	*p*-value	0.04	0.79	0.77	0.56	0.17	0.24
**Barthel Index**	Pearson Correlation	0.26 *	0.14	0.10	0.05	0.13	−0.11
	*p*-value	0.01	0.13	0.29	0.61	0.16	0.25
**TUG**	Pearson Correlation	−0.09	0.01	−0.09	−0.09	−0.09	−0.15
	*p*-value	0.32	0.89	0.34	0.35	0.34	0.12

Abbreviations: NPRS, numerical pain rating scale; WOMAC, Western Ontario and McMaster Universities Osteoarthritis Index; TUG, timed up and go. * Indicates statistical significance *p* < 0.05.
